# Interface-induced collective phase transition in VO_2_-based bilayers studied by layer selective spectroscopy

**DOI:** 10.1038/s41598-025-20752-w

**Published:** 2025-10-21

**Authors:** D. Shiga, S. Inoue, T. Kanda, N. Hasegawa, M. Kitamura, K. Horiba, K. Yoshimatsu, A. F. Santander-Syro, H. Kumigashira

**Affiliations:** 1 Institute of Multidisciplinary Research for Advanced Materials (IMRAM), Tohoku University, Sendai, 980–8577 Japan; 2 Photon Factory, Institute of Materials Structure Science, High Energy Accelerator Research Organization (KEK), Tsukuba, 305–0801 Japan; 3 Université Paris-Saclay, CNRS, Institut des Sciences Moléculaires d’Orsay, 91405 Orsay, France

**Keywords:** Electronic properties and materials, Phase transitions and critical phenomena, Surfaces, interfaces and thin films, Structural properties, Characterization and analytical techniques

## Abstract

We investigated the origin of collective electronic phase transitions induced at the heterointerface between monoclinic insulating VO_2_ and rutile metallic electron-doped VO_2_ layers using in situ soft X-ray photoemission spectroscopy (PES) and X-ray absorption spectroscopy (XAS) on nanoscale VO_2_/V_0.99_W_0.01_O_2_ (001)_*R*_ bilayers. Thanks to the surface sensitivity of PES and XAS, we determined the changes in the electronic structure and V–V dimerization in each constituent layer separately. The layer selective observation of the electronic and crystal structures in the upper VO_2_ layer of the bilayer indicates that the monoclinic insulating phase VO_2_ layer undergoes a transition to the rutile metallic phase by forming the heterointerface. Detailed temperature-dependent measurements reveal that the rutile metallic phase VO_2_ undergoes a transition to the monoclinic insulating phase with a decrease in temperature, as in the case of a VO_2_ single-layer film. Furthermore, during the temperature-induced phase transition in the VO_2_ layer, the spectra are well described by an in-plane phase separation of the rutile metallic and monoclinic insulating phases. These results suggest that the interface-induced transition from the monoclinic insulating to the rutile metallic phase in the VO_2_ layer of bilayers occurs as a collective phase transition derived from the static energy balance between the interfacial energy and the bulk free energies of the constituent layers.

**Supplementary Information:**

The online version contains supplementary material available at 10.1038/s41598-025-20752-w.

## Introduction

Vanadium dioxide (VO_2_) shows an intriguing first-order metal–insulator transition (MIT) near room temperature that has been controversially discussed for over 60 years, particularly because both a structural transition and electron correlations contribute to it^1–17^. Across the MIT, the crystal and electronic structures change from a high-temperature rutile metallic phase to a low-temperature monoclinic insulating phase, concomitant with a dimerization of V atoms along the [001]_*R*_ (*c*_*R*_-axis) direction^2,3^. The MIT is also accompanied by a change in conductivity of several orders of magnitude and exhibits an ultrafast response to external stimuli. Thus, the MIT in VO_2_ has become a central topic in condensed matter physics for its potential applications in Mottronics devices^18–24^. Further, this unusual phenomenon originating from the interplay of lattice dynamics and electron correlations provides a unique testbed to understand the physics of strongly correlated oxides.

The structural phase transition in VO_2_ is characterized by the tilting and pairing of V ions, resulting in the collective dimerization of V ions along the [001]_*R*_ direction in the monoclinic insulating phase^2,3^. Although the MIT that is concomitant with the collective V–V dimerization is reminiscent of the Peierls transition^4,5^, the important role that strong electron correlations have in driving the MIT in VO_2_ has also been evidenced from a large number of experimental and theoretical investigations^6,7^. Therefore, the mechanism of the MIT in VO_2_ is now mainly understood as a cooperative Mott-Peierls transition^9–17^.

This type of MIT in VO_2_ has motivated researchers to control the electronic and structural phase transition by changing the delicate interplay between the electron correlations and the lattice via interfacial effects. Yajima et al.^25^ have reported the occurrence of a collective MIT in bilayer structures composed of VO_2_ layers with different MIT temperatures (*T*_MIT_s). The bilayer consists of an undoped VO_2_ layer with *T*_MIT_ = 293 K and an electron-doped (W-doped) VO_2_ layer with a slightly lower *T*_MIT_ of 257 K. It shows a collective (interlocked) transition at a certain critical layer thickness, while each layer undergoes the MIT at its respective *T*_MIT_ above the collective length, which corresponds to ~ 4.5 nm for each layer. In the collective MIT, the VO_2_ layer undergoes a transition from insulator to metal by forming the interface with the electron-doped VO_2_. This collective transition is interpreted as originating from the static energy balance between the interfacial energy and the bulk free energies (the electronic and structural energies) of the constituent layers based on detailed thermodynamic calculations^25^. In this scenario (Scenario I), the collective phase transition of the VO_2_ layer is interpreted as increased stability of the rutile metallic phase relative to the monoclinic insulating phase to avoid the cost of interfacial energy between the different phases.

In the collective MIT derived from the static energy balance, the MIT in the VO_2_ layer should be the original transition from the monoclinic insulating to the rutile metallic phase. Namely, the metallic state in the VO_2_ layer should be in the rutile metallic phase. However, Lee et al.^26^ have recently suggested the possibility of realizing a new “monoclinic metallic” phase in the VO_2_ layer of the bilayer structure. In this alternative scenario (Scenario II), an isostructural transition from monoclinic insulating to monoclinic metallic phase, namely, a pure electronic phase transition without any structural transition, occurs in the VO_2_ layer adjacent to the electron-doped VO_2_ layer, although the doped layer maintains the rutile metallic phase.

The interface-induced collective phase transition occurring in VO_2_-based bilayers remains an active subject of both experimental and theoretical research^25–28^. However, the lack of conclusive experimental proof regarding which metallic phase actually emerges has hindered the understanding of the phase transition, particularly in theoretical investigations of how interfacial effects can stabilize novel electronic phases or influence the Mott-Peierls transition pathway^27,28^. Therefore, there is a critical need to experimentally clarify the electronic and crystal structures of the VO_2_ layer in the bilayer, while eliminating the influence from the counterpart layer^26^.

To verify which of the two metallic states [rutile metallic phase (Scenario I) or monoclinic metallic phase (Scenario II)] is realized in the VO_2_ layer, it is crucial to observe the electronic and crystal structures in each constituent layer separately. Against this background, in this study, we employed surface-sensitive photoemission spectroscopy (PES) and X-ray absorption spectroscopy (XAS) measurements in the soft X-ray region to investigate the change in the electronic and crystal (characteristic V–V dimer) structures in the top VO_2_ layer of VO_2_/V_0.99_W_0.01_O_2_ (W:VO_2_) (001)_*R*_ bilayer structures. Thanks to a sufficiently shallow probing depth (1.5–2 nm) compared to each layer thickness (4.5 nm) in both spectroscopic measurements^15^, information on the electronic and crystal structures of the upper VO_2_ layer was selectively extracted. The PES and XAS spectra exhibited remarkable changes associated with phase transition in the upper VO_2_ layers: (1) The upper VO_2_ layer exhibits almost the same spectral behavior across the MIT as that of a VO_2_ single-layer film, whereas its *T*_MIT_ is slightly lower than that of the single-layer film. (2) In the metallic states of the upper VO_2_ layer, there is no indication of the V–V dimerization. (3) During the temperature-induced phase transition, both the PES and XAS spectra are described by a linear combination of the rutile metallic and monoclinic insulating phases, indicating the occurrence of an in-plane phase separation. These results strongly suggest that the upper VO_2_ layer undergoes a collective transition from the monoclinic insulating to the rutile metallic phase by forming the heterointerface with the electron-doped VO_2_ layer and that the monoclinic metallic phase does not emerge in the present VO_2_/W:VO_2_ (001)_*R*_ heterostructures. The occurrence of the phase transition from the monoclinic insulating to the rutile metallic phase in the VO_2_ upper layers suggests that the collective phase transition originates from the static energy balance between the interfacial energy and the bulk free energies of the constituent layers (Scenario I).

## Experiment

The VO_2_/V_0.99_W_0.01_O_2_ heterostructures were fabricated on the (001) surface of 0.05 wt% Nb-doped rutile-TiO_2_ substrates in a pulsed-laser deposition (PLD) chamber connected to an in situ PES system at BL-2A MUSASHI of the Photon Factory, KEK^15–17,29,30^. Sintered pellets with appropriate compositions of V_2_O_5_ and V_1.98_W_0.02_O_5_ were used as PLD ablation targets. Each layer was grown at a rate of 0.02 nm s^−1^, as estimated from the Laue fringes in X-ray diffraction (XRD) patterns of a corresponding single-layer film. The growth conditions for each layer are described in detail elsewhere, and the characterization of the heterostructures is given in Supplemental Material^31^. During the deposition, the substrate temperature was maintained at 400 °C, and the oxygen pressure was maintained at 10 mTorr. The thicknesses of the deposited VO_2_ and W:VO_2_ layers, as well as those of the VO_2_ and W:VO_2_ single-layer films, were precisely controlled by deposition time. The surface structures and cleanliness of the heterostructures were confirmed via reflection high-energy electron diffraction (see Fig. S1 in Supplemental Material^31^) and core-level photoemission measurements (see Fig. S3 in Supplemental Material^31^), respectively. The formation of a chemically abrupt interface in the present bilayers was also confirmed by detailed analysis of core-level intensities based on the photoelectron attenuation function (see Fig. S3 in Supplemental Material^31^).

The surface morphologies of the measured heterostructures and single-layer films were analyzed via atomic force microscopy in air (see Fig. S1 in Supplemental Material^31^). The epitaxial relationship and crystalline quality were characterized by XRD, confirming the coherent growth of each layer. A sharp diffraction pattern with well-defined Laue fringes was observed, indicating the high quality of the heterostructures, i.e., homogeneously coherent films with atomically flat surfaces and interfaces (see Fig. S2 in Supplemental Material^31^). All bilayer structures were fabricated under the same conditions as previously reported bilayer structures^25^, wherein chemically abrupt interfaces formed. The sheet conductance was measured using the standard four-probe method with a temperature ramp rate of 10 mK s^−1^.

PES measurements were performed in situ with the use of a VG-Scienta SES-2002 analyzer with a total energy resolution of 120 meV at a photon energy of 700 eV. The vacuum transferring of the grown samples was necessary to prevent the overoxidation of the surface layer^15–17^. The XAS spectra were also measured in situ with linearly polarized light via the measurement of the sample drain current. For linear dichroism measurement of oxygen *K*-edge XAS (O *K* XAS), we acquired the XAS spectra at angles *θ* = 0° and 60° between the [100]_*R*_ direction and the polarization vector ***E*** while maintaining a fixed angle between the direction normal to the interfaces and the incident light (see Fig. S4 in Supplemental Material^31^). Here, we emphasize that the present O *K* XAS measurement is also surface sensitive; its probing depth is estimated to be almost identical to that of the present soft X-ray PES measurements (1.5–2 nm) (see Fig. S5 in Supplemental Material^31^). Therefore, both the PES and XAS spectra reflect the information from almost the same surface region of the top VO_2_ layers. The Fermi energy (*E*_F_) of each sample was determined by the measurement of a gold film that was electrically connected to the sample. As it is common knowledge that VO_2_ exhibits an insulator-to-metal transition upon irradiation by light^35^, we paid particular attention to the possible spectral changes induced by light irradiation (see Fig. S6 in Supplemental Material^31^). The stoichiometry of the constituent layers was carefully characterized by analyzing the relative intensities of the V 2*p* and V 3*p*, O 1*s*, and W 4*f* core levels, confirming that the cation composition of the samples was the same as that of the PLD ablation targets. We carefully carried out temperature-dependent PES and XAS measurements by confirming that the spectral changes with temperature were saturated (hysteresis effects^11,25,32,39^ were no longer present). Furthermore, the sample temperature was maintained at 320 K for half an hour before measuring, and then the measurements were performed only upon cooling to avoid the possible hysteresis effect (see Fig. S7 in Supplemental Material^31^).

## Results and discussion

**Fig. 1:**
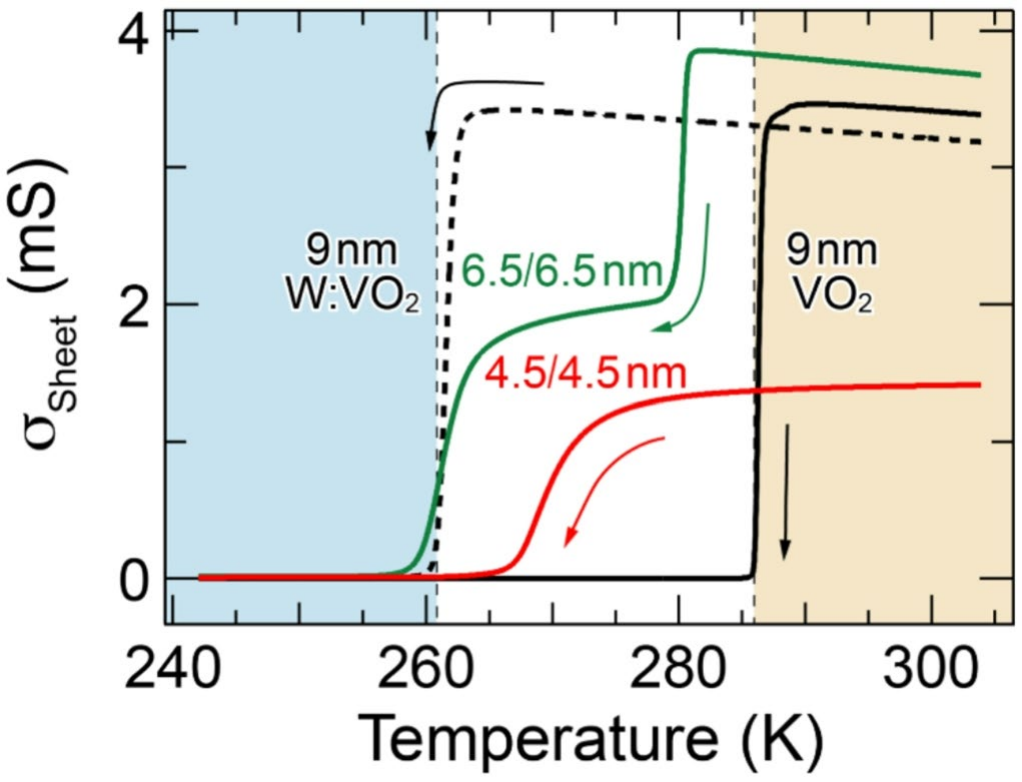
Temperature dependence of σ_Sheet_ upon cooling for VO_2_ (4.5 nm)/W:VO_2_ (4.5 nm) bilayer (red curve) and VO_2_ (6.5 nm)/W:VO_2_ (6.5 nm) bilayer (green curve), together with that of 9 nm VO_2_ and W:VO_2_ single-layer films (solid and dashed black curves, respectively). Vertical dashed lines indicate $T_{\scriptsize\mathrm{MIT}}^{\scriptsize\mathrm{Cool}}$ of the VO_2_ and electron-doped W:VO_2_ single-layer films (286 and 261 K, respectively). Here, $T_{\scriptsize\mathrm{MIT}}^{\scriptsize\mathrm{Cool}}$ is defined as the inflection point in the log_10_σ_Sheet_-*T* curve in the cooling process (see text in more detail). The 6.5 nm bilayer exhibits two-separate transitions, indicating that the upper and lower layers undergo MIT separately. In contrast, the 4.5 nm bilayer shows a single transition, indicating the occurrence of the collective transition in the temperature range of 260–275 K. Note that the behaviors are essentially the same as the previous reports, guaranteeing the comparable quality of the single-layer films and bilayers in the present study^25^. Color shading is a guide for the eye to visually separate specific temperature regions corresponding to the expected electronic phases in the 4.5 nm bilayer, as schematically illustrated in the upper panel of Fig. 2, where both layers are in either the rutile metallic (light orange) or monoclinic insulating (light blue) phase (see text for more details).

Before discussing the PES spectra, we provide evidence that the prepared bilayers show essentially the same properties as those in the previous report^25^. Figure 1 shows the temperature dependence of sheet conductivity σ_Sheet_ upon cooling for VO_2_ (4.5 nm)/W:VO_2_ (4.5 nm) and VO_2_ (6.5 nm)/W:VO_2_ (6.5 nm) bilayers, together with that for 9 nm VO_2_ and W:VO_2_ single-layer films. For the single-layer films, the σ_Sheet_-*T* curve steeply changes across the MIT^15–17,43,44^, as reported previously^25^. The corresponding *T*_MIT_ on the cooling process ($T_{\scriptsize\mathrm{MIT}}^{\scriptsize\mathrm{Cool}}$), which is defined as the inflection point of each log_10_σ_Sheet_-*T* curve, is determined to be 286 K and 261 K for the VO_2_ and W:VO_2_ single-layer films, respectively. The values are in excellent agreement with the previous reports on epitaxial films coherently grown on TiO_2_ (001) substrates, guaranteeing almost the same qualities of the present films as those of the previous ones^15–17,23,25,32,39^. For the bilayers, two separate transitions are observed, reflecting the original $T_{\scriptsize\mathrm{MIT}}^{\scriptsize\mathrm{Cool}}$ of constituent layers. This result suggests that the constituent layers behave independently despite the layered structure, although the $T_{\scriptsize\mathrm{MIT}}^{\scriptsize\mathrm{Cool}}$ corresponding to the VO_2_ layer is slightly lower. In contrast, the two transitions seem to merge at 4.5 nm bilayers, reflecting the occurrence of the collective transition throughout the whole bilayer structure in the temperature range of 260–275 K. The observed layer-thickness dependence is in good agreement with that in the previous study^25^, indicating the achievement of essentially the same properties in the present bilayers. Furthermore, this good agreement indicates the negligible influence of W interdiffusion on the observed phenomena in the present bilayers, since the formation of a chemically abrupt interface has been confirmed by the previous study^25^. Note that our detailed analysis for W 4*f* core-level spectra also supported the formation of a chemically abrupt interface (i.e., the absence of detectable interdiffusion of W ions) in the present study (Fig. S3 in Supplemental Material^31^). It should also be noted that the crystallinity of the measured single-layer films and bilayers is almost identical to that in the previous study^25^ (see Figs. S1, S2^31^).

**Fig. 2:**
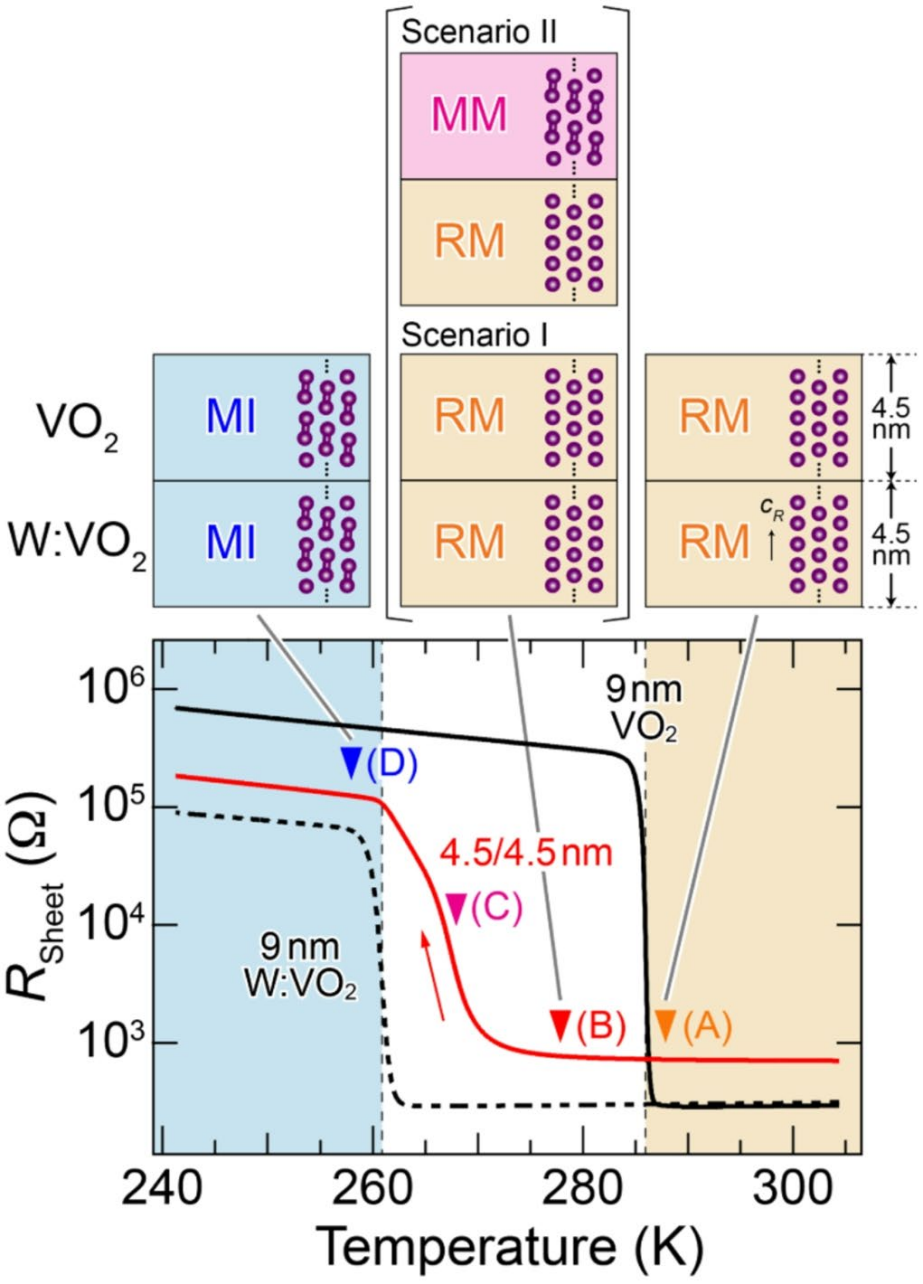
Temperature dependence of *R*_Sheet_ measured upon cooling for VO_2_ (4.5 nm)/W:VO_2_ (4.5 nm) bilayer (solid red curve), along with those of 9 nm VO_2_ and W:VO_2_ single-layer films (solid and dashed black curves, respectively). Vertical dashed lines indicate $T_{\scriptsize\mathrm{MIT}}^{\scriptsize\mathrm{Cool}}$ of the VO_2_ and W:VO_2_ single-layer films (286 and 261 K, respectively), while colored solid triangles indicate the spectroscopic measurement points (*A*–*D*). The upper panel shows the schematic illustration for the expected electronic phases of the bilayer at temperatures *A*, *B*, and *D*. At temperature *B*, the two possible metallic phases of the upper VO_2_ layer based on Scenarios I (interface-induced collective phase transition from monoclinic insulator to rutile metal^25^) and II (isostructural interface-induced collective phase transition from monoclinic insulator to monoclinic metal^26^) are presented. Color shading is a guide for the eye to visually separate specific temperature regions corresponding to the expected electronic phases in the 4.5 nm bilayer, as schematically illustrated in the upper panel, where both layers are in either the rutile metallic (light orange) or monoclinic insulating (light blue) phase (see text for more details). MI, RM, and MM denote the monoclinic insulator, rutile metal, and monoclinic metal, respectively.

To see the behavior of the collective phase transition in more detail, we present the temperature dependence of the sheet resistance *R*_Sheet_ in Fig. 2, alongside reference data of VO_2_ and W:VO_2_ single-layer films. The 4.5 nm bilayer exhibits a single-step MIT at $T_{\scriptsize\mathrm{MIT}}^{\scriptsize\mathrm{Cool}}$ ~ 267 K, in sharp contrast to the two-step MIT in the 6.5 nm bilayer (see Fig. 1). Furthermore, the transition is relatively broad in comparison with the abrupt change across the MIT in the single-layer films, implying a complicated interplay between different phases in the two layers.

According to the previous studies^25,26^, the complicated behaviors may be classified into four temperature regions *A*–*D*, as schematically illustrated in the upper panel of Fig. 2. At temperature *A*, both layers are in the rutile metallic phase, while at temperature *D* both layers are in the monoclinic insulating phase, since at those temperatures the original VO_2_ and W:VO_2_ layers are in the same corresponding rutile metallic or monoclinic insulating phases. Meanwhile, the upper VO_2_ layer shows metallic behavior at temperature *B* as a result of the collective phase transition, although the VO_2_ (W:VO_2_) layer should be the monoclinic insulating (rutile metallic) phase in the case that each of the layers behaves independently as in the 6.5 nm bilayer. In other words, the VO_2_ layer exhibits an insulator-to-metal transition by forming the interface with the electron-doped VO_2_ with lower *T*_MIT_. For Scenarios I and II, the possible metallic phases that emerged at *B* due to the collective interface-induced phase transition are rutile metal and monoclinic metal, respectively. With decreasing the temperature, the metallic phase eventually exhibits a temperature-induced MIT around temperature *C*. As a result, the *T*_MIT_ of the VO_2_ (W:VO_2_) layer is suppressed (enhanced) owing to the proximity effect between the two layers.

**Fig. 3:**
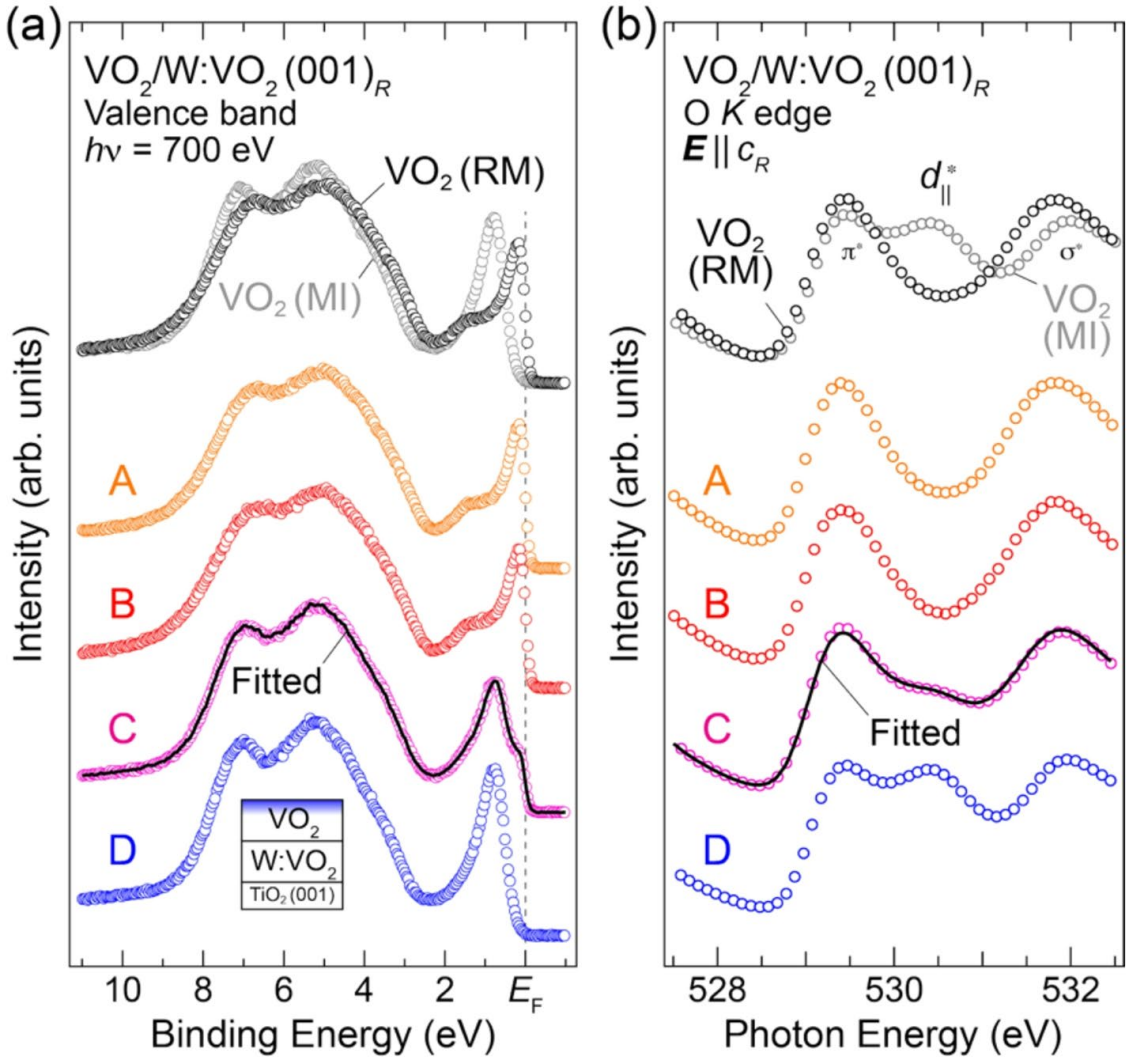
Temperature dependence of in situ (**a**) valence-band spectra and (**b**) O *K* XAS spectra acquired with the ***E*** ∥ *c*_*R*_ geometry^31^ measured upon cooling for VO_2_ (4.5 nm)/W:VO_2_ (4.5 nm) bilayers, together with a VO_2_ film ($T_{\scriptsize\mathrm{MIT}}^{\scriptsize\mathrm{Cool}}$ = 286 K) measured at its rutile metallic and monoclinic insulating phases as references. The measured temperatures of *A*, *B*, *C*, and *D* are shown in Fig. 2. For spectra *C*, the fitted result by the linear combination of the rutile metallic (*A*) and monoclinic insulating (*D*) phases [Eq. (1)] is overlaid by a black solid curve. Note that since the probing depth of the present (**a**) PES and (**b**) XAS measurements are comparable (1.5–2 nm^15,31^), both spectra reflect information on the top VO_2_ layers, as schematically illustrated in the inset in (**a**). The spectra at temperature *B* are almost the same as those of a VO_2_ film in the rutile metallic phase, as well as those at temperature *A*, indicating that the upper VO_2_ layer undergoes the transition from monoclinic insulator to rutile metal by forming the heterointerface. MI and RM denote the monoclinic insulator and rutile metal, respectively.

To identify the metallic phase at temperature *B*, we measured the changes in the electronic structures and V–V dimerization in the upper VO_2_ layer selectively through PES measurements. Figure 3(a) shows the valence-band spectra measured upon cooling at temperatures *A*–*D* for the VO_2_ (4.5 nm)/W:VO_2_ (4.5 nm) bilayers grown on Nb:TiO_2_ (001) substrates, in addition to those of VO_2_ single-layer films in the monoclinic insulating and rutile metallic phases as references. Owing to the surface sensitivity of soft X-ray PES, the spectra mostly reflect the electronic structure of the top 4.5 nm VO_2_ layers, as schematically illustrated in the inset, since the probing depth of the present PES is 1.5–2 nm^15,31^. The spectra contain two main features: structures derived from O 2*p* states at binding energies of 3–10 eV, and peaks derived from the V 3*d* states near *E*_F_. The spectra of the bilayer (i.e., the top VO_2_ layer) exhibit the characteristic features of the MIT in VO_2_ films^11,15–17,41–43^: the spectrum near *E*_F_ at temperature *A* consists of a sharp coherent peak at *E*_F_ and a weak broad satellite around 1.2 eV. Meanwhile, the spectrum at temperature *D* shows a single peak around 0.8 eV, corresponding to the formation of an energy gap at *E*_F_.

For the upper VO_2_ of the bilayer structure, the valence-band spectra at temperatures *A* and *D* are almost exactly the same as those of the VO_2_ single-layer films in the rutile metallic and monoclinic insulating phases, respectively. At temperature *B* where the VO_2_ layer changes from the original monoclinic insulating phase to the metallic phase (rutile metal in Scenario I or monoclinic metal in Scenario II) by forming the heterointerface (see Fig. 2), the spectra exhibit the features representative of the rutile metallic phase of the VO_2_ single-layer film. In fact, the spectrum at temperature *B* is identical to the spectrum at temperature *A* (rutile metallic phase) within the experimental error, suggesting the occurrence of the monoclinic insulator-to-rutile metal transition by forming the interface with the W:VO_2_ layer. The metallic state eventually exhibits the transition to the monoclinic insulating phase at temperature *D* through certain complicated phenomena around temperature *C* (see Fig. 2). Furthermore, focusing on the O 2*p* states, we observe dramatic changes across the temperature-dependent MIT. For VO_2_ films, these changes are attributed to the structural changes (V–V dimerization) concomitant with the MIT in VO_2_^11,15–17^, strongly suggesting the occurrence of temperature-induced structural transition from the rutile metallic phase (temperatures *A* and *B*) to the monoclinic insulating phase (temperature *D*) in the upper VO_2_ layer.

The next crucial issue is whether the crystal structure of the interface-induced metallic phase (see Supplemental Note 7^31^) is the original monoclinic structure with the V–V dimerization or the rutile structure without the dimerization. To determine the crystal structure (monoclinic or rutile) of the interface-induced metallic phase, as well as those of the other phases, we measured, as shown in Fig. 3(b), the polarization dependence of O *K* XAS, which has been previously used as an indicator of V–V dimerization. The O *K* XAS probes the unoccupied O 2*p* partial density of states that are mixed with the unoccupied V 3*d* states and is thus complementary to PES for investigating the electronic structures of conduction bands. Because the V–V dimerization in the monoclinic insulating phase splits the half-filled *d*_∥_ state into occupied *d*_∥_ and unoccupied $d_{\scriptsize{\parallel}}^{*}$ states, an additional peak corresponding to the $d_{\scriptsize{\parallel}}^{*}$ states appears in the XAS spectra only for the monoclinic insulating phase^3,11,30,44–46^. Moreover, owing to strict dipole selection rules, the additional $d_{\scriptsize{\parallel}}^{*}$ states only appear in the spectra acquired with ***E*** parallel to the [001]_*R*_ (*c*_*R*_-axis) direction (***E*** ∥ *c*_*R*_). From the assignments made in previous studies^11,44^, the $d_{\scriptsize{\parallel}}^{*}$ peak emerges at 530.4 eV in the monoclinic insulating phase (temperature *D*) measured with the ***E*** ∥ *c*_*R*_ geometry, whereas it disappears in the rutile metallic one (temperature *A*). The identification of the $d_{\scriptsize{\parallel}}^{*}$ states was further confirmed by the linear dichroism of the XAS spectra: the additional $d_{\scriptsize{\parallel}}^{*}$ peak in the monoclinic insulating phase disappeared for the spectrum taken with ***E*** ⟂ *c*_*R*_ (see Fig. S4 in Supplemental Material^31^). Thus, the existence of the $d_{\scriptsize{\parallel}}^{*}$ peak in the spectra with the ***E*** ∥ *c*_*R*_ geometry can be used as a fingerprint of the V–V dimerization in monoclinic VO_2_^3,11,44,45^. This well-established determination method based on the presence or absence of the $d_{\scriptsize{\parallel}}^{*}$ peak provides a reliable indicator to determine whether the interface-induced phase retains the dimerized monoclinic metallic phase or becomes the nondimerized rutile metallic phase.

Figure 3(b) shows the temperature dependence of in situ O *K* XAS spectra upon cooling acquired with ***E ***∥ *c*_*R*_ of the bilayers. It should be noted that since the probing depth of the present XAS measurements is comparable to that of the PES measurements (Fig. S5 in Supplemental Material^31^), both spectra reflect information on the top 4.5 nm VO_2_ layers [see the inset of Fig. 3(a)]. The spectra for the upper VO_2_ layer show the rutile metallic and monoclinic insulating nature at temperatures *A* and *D*, respectively, as in the case of VO_2_ single-layer films. In addition, the spectral shapes at the two temperatures *A* (rutile metal) and *B* are almost identical, and no detectable $d_{\scriptsize{\parallel}}^{*}$ state is observed, indicating the absence of V–V dimerization at temperature *B*. The selective observation of the electronic and crystal structures indicates that the upper VO_2_ layer undergoes the transition from the monoclinic insulating to the rutile metallic phase by forming the heterointerface (Scenario I).

In the *R*_Sheet_-*T* curve in Fig. 2, the temperature width of the collective MIT for the 4.5 nm VO_2_/W:VO_2_ bilayer is much broader than that of the original VO_2_ and W:VO_2_ single-layer films. Such broadening was also observed in the previous bilayers^25^ as well as the VO_2_ films grown on TiO_2_ (110) substrates^35,47,48^, implying the occurrence of some in-plane phase separation characteristic of VO_2_ nanostructures. To shed light on the phenomena, we measured the PES and XAS spectra at temperature *C*, as shown in Fig. 3. The valence-band spectra at temperature *C* near *E*_F_ exhibit the peak at 0.8 eV, the same binding energy of spectra *D* corresponding to the monoclinic insulating phase, and an additional structure at *E*_F_ indicative of metallic behavior. Upon closer inspection, spectra *C* appear to be an average of the spectra measured at temperatures *A* (rutile metallic phase) and *D* (monoclinic insulating phase). In general, in the case of phase separation, as the size of the soft X-ray light spot used in the present PES and XAS experiments is much larger than the typical size of the phase domains, the measured spectrum is described by a linear combination of the spectra of each phase. Thus, we fit the spectra *I*(*α*) using the following equation^17,49^:

1 $$I\left(\alpha\right)={\alpha}I_{\scriptsize\mathrm{RM}}+\left(1-\alpha\right)I_{\scriptsize\mathrm{MI}},$$

where *α* is the fraction of the rutile metallic phase, and *I*_RM_ and* I*_MI_ are the spectra of the rutile metallic phase (spectra *A*) and monoclinic insulating phase (spectra *D*), respectively. As can be seen at temperature *C*, both the PES and XAS spectra are almost perfectly described by the linear combination of the spectra for *A* (rutile metallic phase) and *D* (monoclinic insulating phase) with *α* = 0.4–0.5. These results indicate that a phase separation of the rutile metallic and monoclinic insulating phases occurs in the upper VO_2_ layer of the VO_2_/W:VO_2_ bilayer structure.

**Fig. 4:**
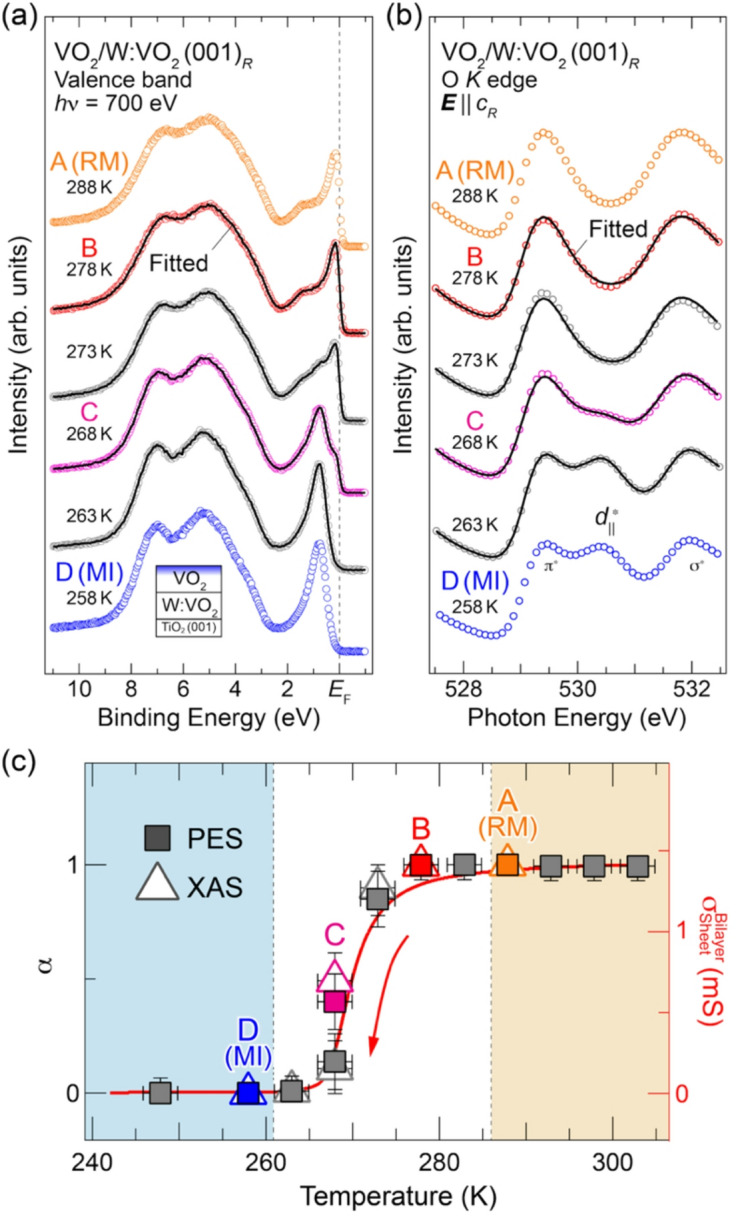
Temperature dependence of in situ (**a**) valence-band and (**b**) O *K* XAS spectra measured near *T*_MIT_ upon cooling for VO_2_ (4.5 nm)/W:VO_2_ (4.5 nm) bilayers. Note that the spectra mostly reflect the electronic structure of the upper VO_2_ layer owing to the surface sensitivity of the present spectroscopic measurements. The fitted results by the linear combination of the rutile metallic (*A*) and the monoclinic insulating (*D*) phases [Eq. (1)] are overlaid on respective spectra. The spectra are almost perfectly described by the linear combination. (**c**) Plot of *α* as a function of temperature. The σ_Sheet_ of the bilayer (Fig. 1) is overlaid as a red curve for comparison. Color shading corresponds to that in Figs. 1 and 2. MI and RM denote the monoclinic insulator and rutile metal, respectively.

To investigate the phase-separation behavior in more detail, we measured the detailed temperature dependence of the PES and XAS spectra across the MIT and fitted them using Eq. (1), as shown in Fig. 4. As can be seen in Fig. 4(a) and (b), all the spectra during the phase transition are well described by the linear combination. The estimated values of *α* are plotted as a function of measured temperature in Fig. 4(c), together with the corresponding σ_Sheet_ (Fig. 1). Although the electrical conductance in the case of phase separation must be considered in terms of a percolation model, the good agreement between the σ_Sheet_ and *α* values suggests that the complex behavior across the MIT in the bilayer can be attributed to the phase separation. Furthermore, considering the difference in probing depth between these two types of measurements (soft X-ray spectroscopies are sensitive to the top VO_2_ layer, while resistivity measurement probes the entire bilayer), it is likely that the phase domain structures of the upper and lower layers in the bilayers are the same and that the bilayers undergo the collective phase transition. In such a case where the separation of the rutile metallic and monoclinic insulating phases occurs, it may appear that the monoclinic metallic phase emerges. Therefore, it is important to investigate the electronic and V–V dimer structures of each layer selectively using spectroscopic techniques that can prove only specific regions, as in this study.

The selective observation of the electronic and crystal structures of the constituent layers in the bilayers indicates that the upper VO_2_ layer undergoes the transition from the monoclinic insulating to the rutile metallic phase by forming the heterointerface between VO_2_ and W:VO_2_ layers. The significance of this study is the experimental demonstration of a collective monoclinic insulating-to-rutile metallic phase transition induced in the VO_2_/W:VO_2_ heterostructures. By providing experimental validation for the proposed mechanism^25^, the present study offers a solid basis for the thermodynamic modeling approach (including calculations of the free energy) and reveals the importance of such energy balance in bilayer systems. These results therefore establish a solid groundwork for future quantitative understanding of VO_2_-based heterostructures. However, it should be bear in mind that the present study does not eliminate the possibility of the emergence of the monoclinic metallic phase in VO_2_-based bilayer structures. In comparison to the previous results of Lee et al.^26^, there are several differences from the bilayers examined here: the difference (7 K) of *T*_MIT_ between constituent layers is much smaller than that in the present case (25 K), and electrons are doped by oxygen vacancies (VO_2−δ_). The significantly smaller difference in *T*_MIT_ reflects a closer proximity between the electronic and structural energies of constituent layers, which may induce the emergence of the new equilibrium monoclinic metallic phase in the VO_2_/VO_2−δ_ bilayer^26^. Furthermore, in chemically doped VO_2_, the V–V dimer structure is known to change significantly depending on the type and concentration of the dopant, resulting in the complicated electronic phase diagram^32,50,51^. Therefore, the resultant complicated static energy balance between the interfacial energy and the bulk free energies of constituent layers may lead to the emergence of the monoclinic metallic phase under certain conditions. To gain a better understanding of the interface-induced collective phenomena occurring in the VO_2_-based bilayers, further systematic investigations are required. In particular, investigations of the more detailed dopant and layer-thickness dependencies are necessary. Complementary probes such as temperature-dependent Raman spectroscopy may offer additional insights into the interplay between the electronic and structural transitions^52,53^. Also, further theoretical works that adequately treat these effects will be necessary to examine the possibility of a new electronic phase in the bilayers.

## Conclusion

We performed in situ PES and XAS measurements on VO_2_/W:VO_2_ bilayers to investigate the changes in the electronic structure and characteristic V–V dimerization across the collective phase transition induced at the heterointerface between the monoclinic insulating phase VO_2_ and rutile metallic phase W:VO_2_ layers. Thanks to the surface sensitivity of PES and XAS in the soft X-ray region, we separately extracted the changes in the top VO_2_ layer. The spectra exhibited remarkable change associated with the collective phase transition: (1) The upper VO_2_ layer exhibits almost the same spectral behavior across the MIT as that of a VO_2_ single-layer film, whereas its *T*_MIT_ is slightly lower than that of the single-layer film. (2) In the metallic states of the upper VO_2_ layer, there is no indication of the V–V dimerization. (3) During the phase transition, both the PES and XAS spectra are described by a linear combination of the rutile metallic and monoclinic insulating phases, indicating the occurrence of in-plane phase separation. These results strongly suggest that the upper VO_2_ layer undergoes a collective transition from the monoclinic insulating to the rutile metallic phase by forming the heterointerface with the electron-doped VO_2_ layer. The occurrence of the phase transition from the monoclinic insulating to rutile metallic phase in the VO_2_ upper layers suggests that the collective phase transition originates from the static energy balance between the interface energy and the bulk free energies of the constituent layers.

## Supplementary Information

Below is the link to the electronic supplementary material.

Supplementary Material 1

## Data Availability

The datasets generated and/or analyzed during the current study are available from the corresponding author upon reasonable request.
