# Supplementary material for: Interface-induced collective phase transition in VO2-based bilayers studied by layer selective spectroscopy
Source: Sci Rep. 2025 Oct 21;15:36743. doi: 10.1038/s41598-025-20752-w (PMC12540892; doi:10.1038/s41598-025-20752-w)
Supplement: Supplementary file 1 — Supplementary Material 1 [file 41598_2025_20752_MOESM1_ESM.pdf]

Supplemental Material

Interface-induced collective phase transition in VO_2-based bilayers studied by layer selective spectroscopy

D. Shiga[1,2,*], S. Inoue[1], T. Kanda[1], N. Hasegawa[1], M. Kitamura[2], K. Horiba[2], K. Yoshimatsu[1], A. F. Santander-Syro[3], and H. Kumigashira[1,2,*]

[1] *Institute of Multidisciplinary Research for Advanced Materials (IMRAM), Tohoku University, Sendai 980–8577, Japan*

[2] *Photon Factory, Institute of Materials Structure Science, High Energy Accelerator Research Organization (KEK), Tsukuba 305–0801, Japan*

[3] *Institut des Sciences Moléculaires d'Orsay, Université Paris-Saclay, 91405 Orsay, France*

[*]Correspondence authors: dshiga@tohoku.ac.jp, kumigashira@tohoku.ac.jp

Supplemental Note 1. Sample characterization

A. Surface crystallinity and morphology

The surface structures and cleanliness of all the measured samples — 9 nm VO_2 and $V_{0.99}W_{0.01}O_2$ (W:VO_2) single-layer films and VO_2 (4.5 nm)/W:VO_2 (4.5 nm) bilayer structures grown on TiO_2 (001) substrates — were confirmed via *in situ* reflection high-energy electron diffraction (RHEED). Typical *in situ* RHEED patterns are shown in the left panel of Fig. S1. The integer-order Bragg spots clearly appear at the same positions as those of the bare TiO_2 surface as indicated by vertical dashed lines. Furthermore, clear Kikuchi lines are also observed in the samples and substrates. These results indicate good surface crystallinity and cleanliness of the bilayers, as well as coherent epitaxial growth on the substrates.

The atomically flat surfaces of all the measured samples were confirmed by *ex situ* atomic force microscopy (AFM). The corresponding AFM images are shown in the right panel of Fig. S1. The AFM images show similar surface morphologies to that of the original TiO_2 substrate. The root-mean-square roughness R_{rms} estimated from the AFM images is all less than 0.2 nm. These values are almost the same as that of the original TiO_2 substrate (R_{rms} = 0.16 nm). The R_{rms} values of the measured films were all less than the V-V dimer length (approximately 0.3 nm), indicating that these films were controlled to the scale of the V-V dimer length and that the smooth surface and interface were maintained not only in the single-layer films but also in the bilayer structures.

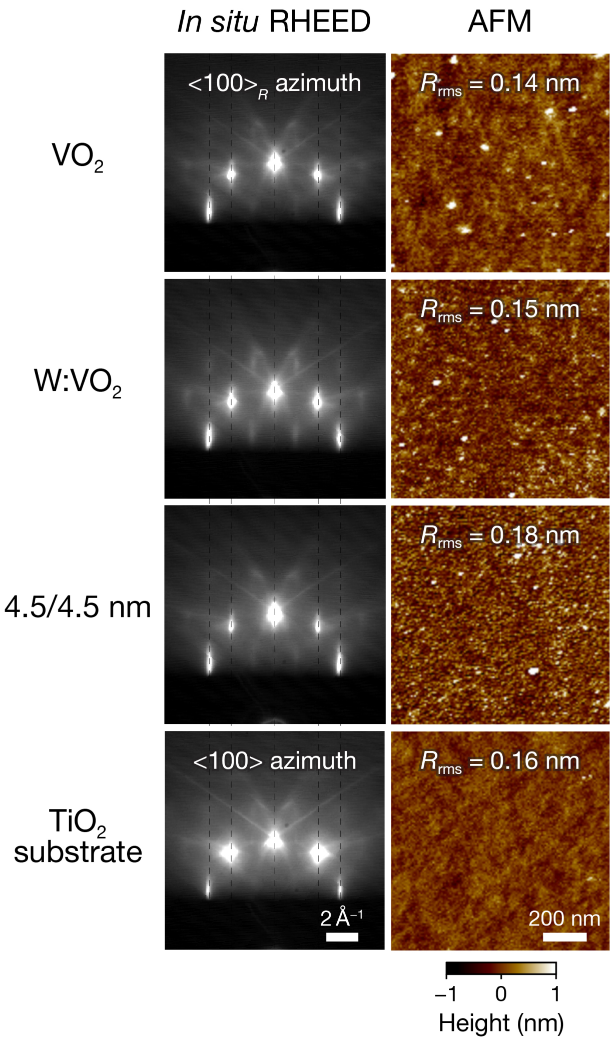

Fig. S1. Typical *in situ* RHEED patterns (0th Laue zone) captured along the <100>$_R$ azimuthal direction (left panel) and the corresponding AFM images (right panel) of the measured 9 nm VO_2 single-layer films, 9 nm W:VO_2 single-layer films, and VO_2 (4.5 nm)/W:VO_2 (4.5 nm) bilayer structures grown on TiO_2 (001) substrates, along with those of the substrate as references.

B. Crystal structure

The crystal structures of the 9 nm VO_2 and $V_{0.99}W_{0.01}O_2$ (W:VO_2) single-layer films and the VO_2 (4.5 nm)/W:VO_2 (4.5 nm) bilayer structures were characterized by x-ray diffraction (XRD) measurements, which confirmed the achievement of single phase in both the single-layer films and the absence of another phase in the bilayer, as well as their coherent growth on TiO_2 (001) substrates. Figure S2(a) shows the out-of-plane XRD patterns around the $(002)_R$ reflection for

the films and bilayer. The formation of single phase for the films and the absence of another phase in the bilayer are confirmed. In addition, the presence of well-defined Laue fringes indicates the formation of atomically flat surfaces and chemically abrupt interfaces. The estimated out-of-plane lattice constant (c_R) of the VO_2 and W:VO_2 films are 0.2829(4) nm and 0.2839(4) nm, respectively. These values are in good agreement with previous reports [32], guaranteeing the high quality of the present samples. The XRD pattern for the bilayer is well fitted with a two-layer model consisting of a VO_2 layer [c_R = 0.2829(4) nm] and a W:VO_2 layer [c_R = 0.2839(4) nm], indicating the formation of a chemically abrupt heterointerface between the two layers, as well as between the layer and the substrate, as reported previously [25]. The coherent growth of the bilayer on TiO_2 (001) substrates is confirmed by the reciprocal space mapping (RSM) around the $(112)_R$ reciprocal point, as well as the absence of another phase, as shown in Fig. S2(b). As shown in Fig. S2(b), the in-plane lattice constant of the layers remains pinned to that of the TiO_2 substrate, indicating the coherent growth of the bilayer. These crystallographic results identify the present films and bilayers as being highly crystalline.

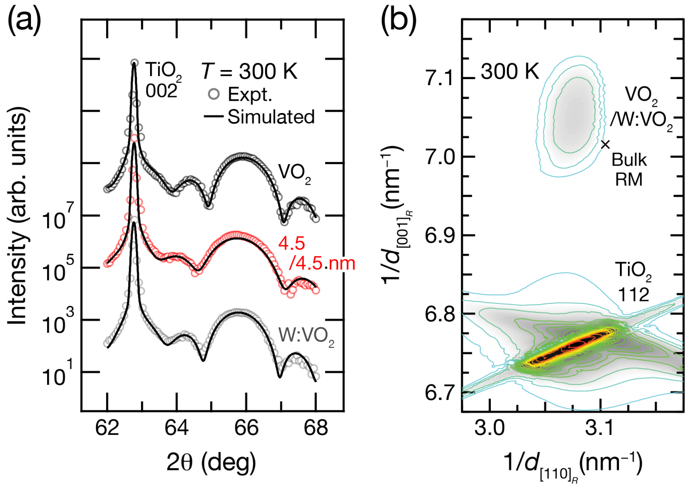

Fig. S2. (a) Typical out-of-plane XRD patterns around the $(002)_R$ reflection measured at room temperature for 9 nm VO_2 and W:VO_2 single-layer films and VO_2 (4.5 nm)/W:VO_2 (4.5 nm) bilayers grown on TiO_2 (001) substrates. Simulated XRD patterns based on corresponding single- or two-layer models (black curves) are overlaid. (b) RSM around the $(112)_R$ reciprocal point for the bilayer. The cross mark denotes lattice constants of bulk VO_2 in the rutile metallic (RM) phase [33] for reference.

Supplemental Note 2. Core-level photoemission measurements

A. Chemical states of V ions

Figure S3(a) shows the *in situ* V 2*p* core-level spectrum measured in the monoclinic insulating phase for the 4.5 nm bilayer structures, alongside hard x-ray photoemission (HAXPES) spectra for VO_2 single-layer films as a reference for the V^{4+} state [15]. The V 2*p* core-level spectrum of the bilayer is almost identical to that of V^{4+}. The excellent agreement indicates that the V ions in the bilayers exist in a tetravalent state.

B. Formation of a chemically abrupt interface

Figure S3(b) shows the *in situ* V 3*p* and W 4*f* core-level spectra for the 6.5 and 4.5 nm bilayers, as well as the 9 nm W:VO_2 single-layer films. Although the doping concentration is only 1at% ($x = 0.01$ in $V_{1-x}W_xO_2$), prominent W 4*f* peaks are clearly observed in the 9 nm W:VO_2 single-layer films due to the sharpness and large photoionization cross section of the W 4*f* states. This enables us to evaluate the degree of interdiffusion of W ions in the bilayer structures using the attenuation of the W 4*f* signal. As can be seen in Fig. S3(b), with increasing the undoped VO_2 overlayer thickness (t), the intensity of W 4*f* states ($I_{W\,4f}$) steeply reduces and almost disappears at $t = 6.5$ nm, owing to the attenuation of photoelectrons emitted from the buried W:VO_2 layer by the VO_2 overlayer. This steep attenuation behavior strongly suggests the formation of a chemically abrupt interface. To evaluate the chemical abruptness of the interfaces, we plot $I_{W\,4f}$ as a function of t in the inset of Fig. S3(b) and compare it with a photoemission attenuation function calculated using the TPP-2M code [34]. The excellent agreement between the two indicates the formation of a chemically abrupt interface (i.e., negligible interdiffusion of W ions) in the present bilayers.

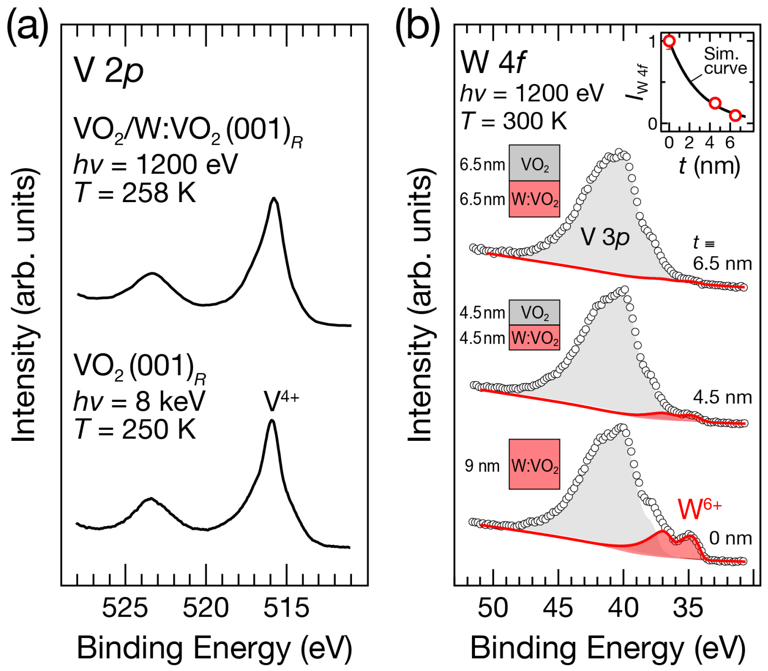

Fig. S3. (a) *In situ* V 2*p* core-level spectrum measured at $T = 258$ K (insulating phase) for the VO_2 (4.5 nm)/W:VO_2 (4.5 nm) bilayer structures, alongside HAXPES spectrum for VO_2 single-layer films as a reference for the V^{4+} state [15]. (b) V 3*p* and W 4*f* core-level spectra for the 6.5 nm and 4.5 nm bilayers and 9 nm W:VO_2 single-layer films. Each spectrum is fitted with Voigt functions corresponding to the relevant core levels after subtracting the background using a Shirley function. Inset shows a plot of $I_{W\,4f}$ (red open circles) as a function of *t*, in comparison with a calculated photoemission attenuation curve (black line) based on the TPP-2M code [34]. The excellent agreement between the two indicates the formation of a chemically abrupt interface in the present bilayers.

Supplemental Note 3. Experimental geometry in polarization-dependent x-ray absorption measurement

Figure S4 depicts the schematic of our experimental geometry for *in situ* polarization-dependent x-ray absorption spectroscopy (XAS) measurements, including the crystal axes of a VO_2 $(001)_R$ film sample and the polarization vector $\boldsymbol{E}$. Regarding linear dichroism measurements for XAS, we acquired the spectra at angles $\theta = 0°$ and 60° between $\boldsymbol{E}$ and the a_R-axis direction, which is defined as the *a*-axis direction in the rutile structure ($[100]_R$), while maintaining a fixed angle of 60° between the direction normal to the $(001)_R$ surface and the incident light. The maintenance of the fixed angle between the direction normal to the surface and the incident light ensures that the probing depth corresponding to the two spectra with different θ values is the same. In the present experimental geometry, XAS spectra with $\boldsymbol{E} \parallel c_R$ ($I_{\parallel}$) can be deduced from the expression $I_{\parallel} = (4/3)(I - I_{\perp}/4)$, where I and $I_{\perp}$ (namely, that corresponding to $\boldsymbol{E} \perp c_R$) denote XAS spectra measured with grazing ($\theta = 60°$) and normal ($\theta = 0°$) incidences, respectively.

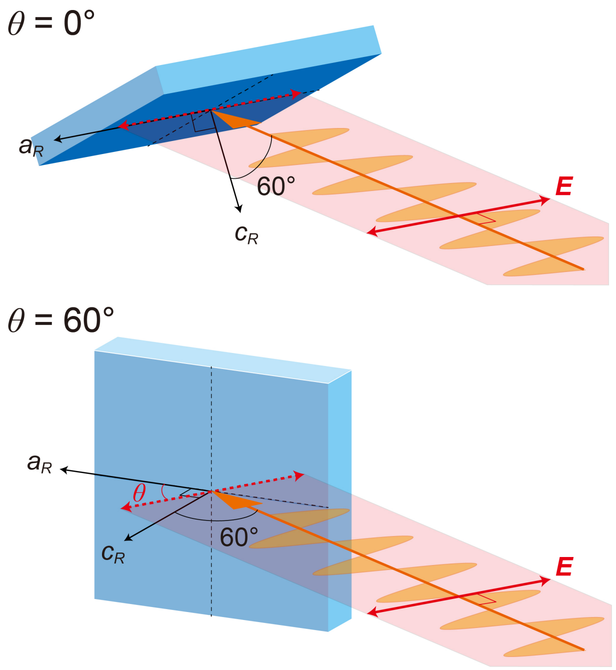

Fig. S4. Schematic of our experimental geometry of polarization-dependent XAS measurements of a VO_2 $(001)_R$ film at $\theta = 0°$ and 60°. The a_R- and c_R-axis directions are defined as the *a*- and *c*-axis directions in the rutile structure, respectively.

Supplemental Note 4. Probing depths of the present soft x-ray spectroscopies

To evaluate the probing depth of photoemission spectroscopy (PES) λ^{PES} and x-ray absorption spectroscopy (XAS) λ^{XAS}, we measured the overlayer-thickness dependence of Ti 2*p* core-level spectra and Ti L_3-edge XAS for VO_2/Nb:TiO_2 (001) single-layer films, as shown in Figs. S5(a) and S4(b), respectively. Each spectrum is normalized to the incident photon flux; hence, the observed intensity reduction with increasing VO_2 overlayer thickness *t* reflects the attenuation of the Ti-derived signal from buried TiO_2 substrates by the VO_2 overlayer. For both the soft x-ray (SX) spectroscopies, the intensity decreases steeply with increasing *t* and becomes almost undetectable at $t = 3$ nm, highlighting the surface sensitivity of the two spectroscopic measurements, as well as the formation of the chemically abrupt interface between the VO_2 layer and TiO_2 substrate [15]. To evaluate the probing depths for PES and XAS, we plot the Ti 2*p* core-level intensity $I_{\mathrm{Ti}\,2p}^{\mathrm{PES}}$ and Ti L_3 XAS intensity $I_{\mathrm{Ti}\,2p}^{\mathrm{XAS}}$ as a function of *t* in Fig. S5(c) and fit them by attenuation functions of $I_{\mathrm{Ti}\,2p}^{\mathrm{PES}} = e^{-t/\lambda^{\mathrm{PES}}}$ for PES and $I_{\mathrm{Ti}\,2p}^{\mathrm{XAS}} = e^{-t/\lambda^{\mathrm{XAS}}}$ for XAS, respectively. As shown in Fig. S5(c), the intensity reductions are well fitted to the attenuation functions with $\lambda^{\mathrm{PES}} = 0.55(2)$ and $\lambda^{\mathrm{XAS}} = 0.70(1)$. The similar values of λ^{PES} and λ^{XAS} indicate that both SX spectroscopies probe approximately the same region. Based on these values, the signal contributions from the upper 4.5 nm thick VO_2 layer in the present PES and XAS measurements are estimated to be 99.97% and 99.84%, respectively. These results indicate that both PES and XAS measurements predominantly probe the upper VO_2 layer of bilayer structures.

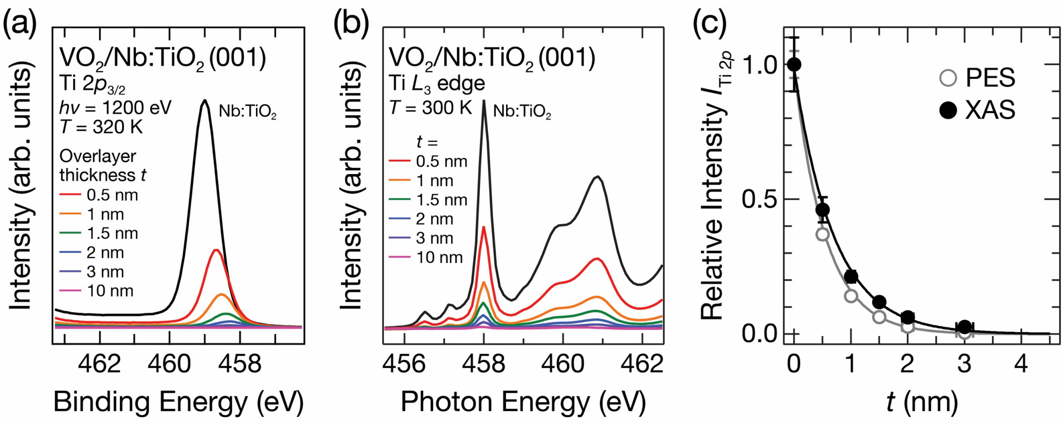

Fig. S5. (a) Ti 2$p_{3/2}$ core-level spectra [15] and (b) Ti L_3 XAS spectra for VO_2/Nb:TiO_2 (001) single-layer films with varying *t*, along with those of the Nb:TiO_2 substrate as references. (c) Relative intensities of the Ti 2$p_{3/2}$ core-level PES spectra ($I_{\mathrm{Ti}\,2p}^{\mathrm{PES}}$, gray open circles) and Ti L_3

XAS spectra ($I_{\mathrm{Ti}\,2p}^{\mathrm{XAS}}$, black solid circles), plotted as functions of *t*. Gray and black curves represent fits using attenuation models with λ^{PES} = 0.55(2) nm and λ^{XAS} = 0.70(1) nm, respectively.

Supplemental Note 5. Irradiation-time dependence of valence band spectra

It is well known that VO_2 exhibits an insulator-to-metal transition upon irradiation by soft x-ray (SX) [35]. Therefore, to perform the photoelectron spectroscopic measurements within a period in which no detectable spectral changes due to SX irradiation are observed, we measured the irradiation-time dependence of the valence-band spectra for insulating VO_2, as shown in Fig. S6. No detectable spectral differences near the Fermi level (E_F) are observed between the spectra acquired before (initial) and after 15 minutes of SX irradiation, whereas certain slight changes are detectable in the spectra after 45 minutes. Therefore, we changed the location of the light spot on each sample every 15 minutes during the present measurements. All the PES and XAS spectra shown in the text and Supplemental Material were acquired within this period. Thus, the light irradiation effects are negligible in the present study.

As shown in Fig. S6, the intensity (area under the curve) of the O 2*p* bands decreases after 45 minutes of SX irradiation in parallel with the broadening of the V 3*d* band near E_F, whereas the area under the curve for the V 3*d* band remains unchanged. This indicates that the changes in the V 3*d* spectra near E_F might originate from oxygen vacancies generated by SX irradiation, as observed in many transition metal oxide systems [35–38].

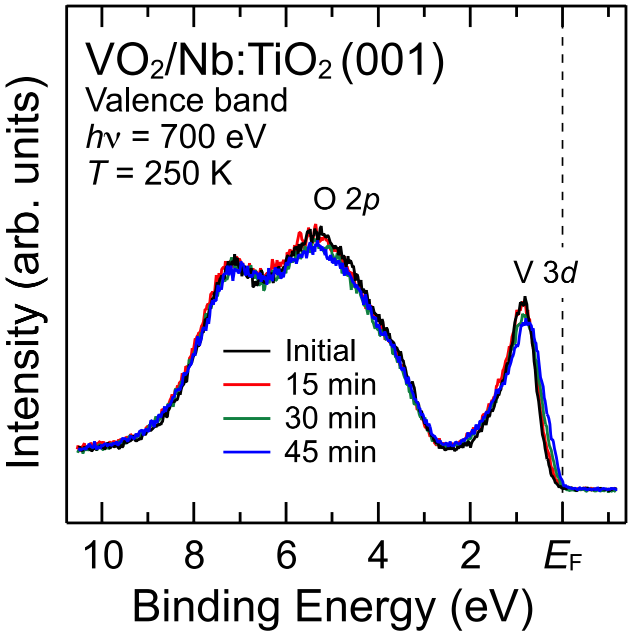

Fig. S6. Irradiation-time dependence of valence-band spectra for VO_2/Nb:TiO_2 (001) films.

Supplemental Note 6. Thermal hysteresis in sheet resistance

Figure S7 shows the temperature dependence of the sheet resistance R_{Sheet} for VO_2 (4.5 nm)/W:VO_2 (4.5 nm) bilayers, along with 9 nm epitaxial VO_2 and W:VO_2 single-layer films. Note that the R_{Sheet}-T curves measured upon cooling are the same as those in Fig. 2 in the main text. The hysteresis loop characteristic of a first-order transition in VO_2 films is clearly observed for the bilayer. The hysteresis loops almost close at 300 K. Therefore, to avoid the possible hysteresis effects in the present spectroscopic measurements, the sample temperature was maintained at 320 K for half an hour prior to the spectroscopic measurements, and then all spectroscopic data were acquired only during cooling.

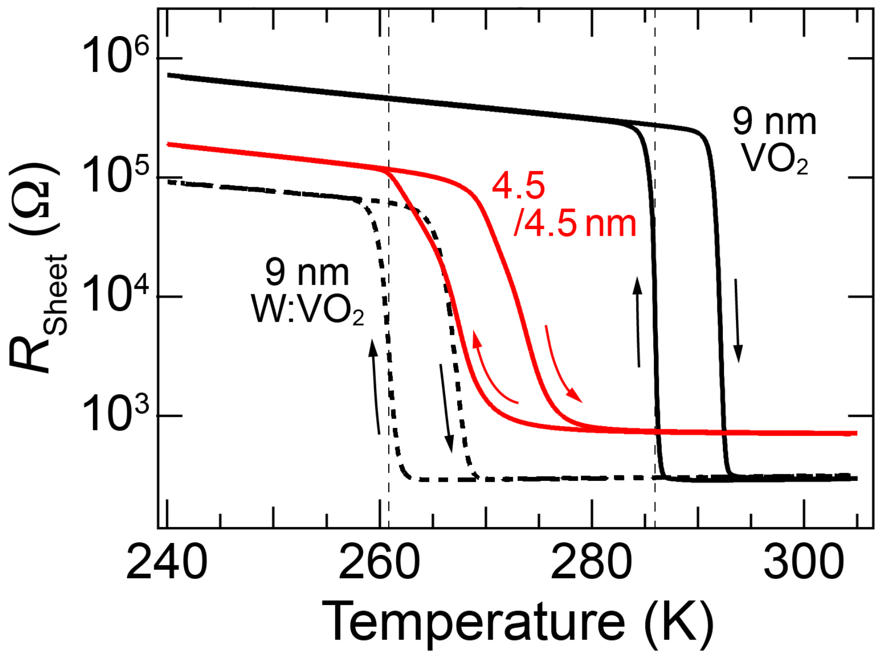

Fig. S7. Temperature dependence of R_{Sheet} for 4.5/4.5 nm bilayer, which consists of VO_2 (4.5 nm) on W:VO_2 (4.5 nm) (solid red curve), and 9 nm epitaxial VO_2 and W:VO_2 single-layer films (solid and dashed black curves, respectively) grown on TiO_2 (001) substrates. Vertical dashed lines indicate $T_{\mathrm{MIT}}^{\mathrm{Cooling}}$ of the VO_2 (286 K) and electron-doped W:VO_2 (261 K) single-layer films.

Supplemental Note 7. Definition of the interface-induced transition

In the present bilayer structures, we observed that the upper VO_2 layer, which is originally in the monoclinic insulating phase, undergoes a transition to the rutile metallic phase upon forming the interface with the W:VO_2 layer at temperature *B*, as schematically illustrated in the inset of Fig. 2. In this context, the phase transition is not triggered by temperature but rather driven by interface effects. In other words, temperature merely serves as an external tuning parameter to observe the changes induced by interface effects, but it is not the critical driving factor for the interface-induced phase transition. Therefore, although the observed phenomena can indeed be considered an integrated effect of both temperature- and interface-driven transitions, we define it as an "interface-induced transition" in a narrow sense, to distinguish it from the usual temperature-driven transition.
